# Zap-X Radiosurgery for Skull Base Meningiomas: A Long-Term Follow-Up Case Report

**DOI:** 10.7759/cureus.86427

**Published:** 2025-06-20

**Authors:** Jingmin Bai, Jinyuan Wang, Chengcheng Wang, Xiangkun Dai, Wei Yu, Longsheng Pan, Baolin Qu

**Affiliations:** 1 Department of Radiation Oncology, The First Medical Center of the Chinese People’s Liberation Army (PLA) General Hospital, Beijing, CHN; 2 Department of Neurosurgery, The First Medical Center of the Chinese People’s Liberation Army (PLA) General Hospital, Beijing, CHN

**Keywords:** long-term follow-up, meningiomas, neurosurgery, radiosurgery, zap-x

## Abstract

This article reports two cases of meningioma patients treated with the novel radiosurgical device ZAP-X stereotactic radiosurgery. During the long-term follow-up period of up to four years after treatment, neither patient developed new neurological deficits, and no adverse reactions of Common Terminology Criteria for Adverse Events grade 2 or above occurred. By retrospectively analyzing the clinical diagnosis, treatment processes, and follow-up results of these two patients, this study aims to provide practical references for the large-scale clinical application of this technology.

## Introduction

Meningiomas are one of the most common primary tumors of the central nervous system, accounting for 38% of intracranial tumors, with approximately 80% being benign (WHO grade I) [1]. Surgical gross total resection is the preferred method for curing benign meningiomas. However, due to the location of the tumor (such as deep regions like the skull base) and the limitation of adjacent neurovascular structures, radical resection is difficult to achieve in about 30% of cases [2]. For these patients, stereotactic radiosurgery (SRS) and fractionated stereotactic radiotherapy (SRT) have become important alternatives or adjuvant treatment options after surgery. Existing evidence-based medical evidence shows the five-year local control rate of SRS/SRT for meningiomas can reach 91%-100%, and the 10-year control rate is 69.6%-89.9% [3], confirming the key role of radiosurgery in tumor control.

Gammaknife, Cyberknife, and linac-based radiosurgery techniques are commonly used for the definitive management of meningiomas. Although traditional SRS systems have been widely used, they have inherent limitations, such as non-uniform dose distribution caused by isocenter position for tumors with complex shape, reliance on external shielding facilities, increased costs for treatment room construction, and the inability to deliver fractionated treatments with frame-only based GK systems due to their dependence on rigid frame fixation. In recent years, the ZAP-X stereotactic radiosurgery system (ZAP Surgical Systems, USA), based on robotics technology, has provided an innovative solution to address these issues [4]. This system integrates a 3 MV linear accelerator with self-shielding design, combined with eight tungsten alloy collimators (diameter 4-25 mm) and a 450 mm source-to-axis distance, enabling submillimeter precision for single-fraction or fractionated treatments. The ZAP-X system uses a dual-axis gantry combined with an automatically rotating tungsten wheel collimator, providing thousands of non-coplanar irradiation directions. The penumbra of ZAP-X is superior to traditional SRS systems, significantly reducing the radiation dose to organs at risk (OARs) such as the optic nerve and brainstem [5]. Meanwhile, the system integrates an MV detector to monitor the output dose of each beam in real-time, ensuring the dose verification and treatment safety. Additionally, the innovative compact self-shielding effectively reduces the cost and installation of the treatment room [6].

Early clinical studies have preliminarily verified the effectiveness and safety of the ZAP-X system. Horiba et al. reported that six patients with brain tumor recurrence after Gamma Knife treatment were treated with ZAP-X, achieving a six-month progression-free survival rate of 100%, with only one case showing asymptomatic imaging changes [7]. However, there is still a lack of high-level evidence on the long-term efficacy of ZAP-X. This study analyzes the treatment data of two patients with meningiomas in complex locations adjacent to the brainstem, combined with dosimetric parameters and four-year follow-up results, to explore the clinical application value of the ZAP-X system.

## Case presentation

Basic patient information

Case 1

A 38-year-old female patient presented with sudden left facial muscle clonus while eating in June 2018, which resolved spontaneously after several minutes. Enhanced cranial MRI revealed a left petrous apex meningioma with imaging features consistent with meningioma. Based on this diagnosis, she underwent resection of the petrous apex tumor via a left subtemporal approach under general anesthesia in November 2018, and the postoperative pathology was confirmed as meningioma (WHO grade I). Two years after surgery (October 2020), she developed vestibular vertigo without obvious inducement, and the follow-up contrast-enhanced cranial MRI showed tumor recurrence in the original surgical area. To control the progression of the recurrent lesion, after full communication with the patient and her family, SRS with ZAP-X was performed on the recurrent lesion in December 2020.

Case 2

A 65-year-old female patient presented with dizziness and occipital-cervical radiating pain without an obvious cause in August 2020, which worsened significantly during the day. Enhanced cranial MRI showed a space-occupying lesion in the foramen magnum area. She underwent tumor resection via a left far-lateral approach under general anesthesia in September 2020. Due to intraoperative assessment that complete tumor resection would cause irreversible damage to the vagus nerve and accessory nerve complex, R2 subtotal resection was performed. Postoperative pathology confirmed meningioma (WHO grade I). Postoperatively, she developed sequelae including left vocal cord paralysis (vagus nerve injury) and mild left limb paresis (corticospinal tract involvement). To control the progression of the residual lesion, after full communication with the patient and her family, salvage radiotherapy with ZAP-X SRS was performed in April 2021.

Simulation

The patients were positioned supine with a thermoplastic head mask using the ZAP-X dedicated positioning plate. A Siemens SOMATOM Definition AS CT simulator was used with a slice thickness of 1 mm, mAs of 300, tube voltage of 120 kV, field of view of 300 mm, and matrix of 512 × 512. The upper boundary included the top of the head mask, and the lower boundary avoided scanning the CT couch. Due to the specificity of the ZAP-X treatment couch, special positioning requirements were applied. The same position with CT simulation was maintained in the MRI simulation with the United Imaging Omega MR simulator (1 mm slice thickness, T1+C sequence). To improve patient comfort during the long treatment time, three layers of soft bubble pads were applied on top of the positioning headrest before shaping the head mask, further enhancing the fixation accuracy.

Delineation, prescription, and OAR dose constraints

Simulation CT and MR images were imported into MIM Maestro (version 6.9.5) for image fusion. Target and OARs were contoured according to published consensus guidelines [8]. For Case 1, the gross target volume (GTV) was the left petrous apex recurrent lesion, and for Case 2, the GTV was the left foramen magnum residual lesion. The planning target volume (PTV) was uniformly expanded by 1 mm of the GTV, avoiding OARs and anatomical barriers. OARs included brain tissue, brainstem, left/right optic nerve, left/right eye, left/right lens, left/right cochlea, spinal cord, and optic chiasm. Both cases received a prescription dose of 19.5 Gy in three fractions. Dose constraints for OARs [9,10] were brainstem: Dmax < 23.1 Gy, V15.9 < 0.5 cc; optic pathway (including the optic nerves and chiasm): Dmax < 17.4 Gy, V15.3 < 0.2 cc; cochlea: Dmax < 17.10 Gy, and the dose to the lens and eyes was minimized. CT image and RT structures were transferred to the ZAP-X treatment planning system for plan design.

Treatment planning and dosimetry

A physicist with 10 years of experience designed plans with the principle of PTV coverage ≥95% and individualized OAR dose limitations. To reduce the risk of tumor cystic transformation from central high-dose regions, the prescription dose was normalized to 70% of the total dose.

During the treatment planning design, an individualized treatment pathway for each patient was first generated. By placing a 25 mm collimator isocenter at the geometric center of the PTV in the simulation CT, a dedicated path-planning software was used to create the individualized treatment pathway. This design process aimed to ensure a safe distance between the collimator and the patient’s body while dynamically adjusting the density of path nodes based on the complexity of the target morphology, and to optimize the treatment pathway, ensuring the precise implementation of the treatment plan and the achievement of clinical goals.

Subsequently, the treatment plan was updated to the optimized individualized pathway. Based on the three-dimensional morphological characteristics of the target volume, isocenters were manually placed, and the initial conformal coverage of the target volume was achieved by adjusting the collimator size for each isocenter. According to our institution’s planning experience, larger collimators were first placed to cover the main portion of the target, and smaller collimators fill in the dose-deficient areas [11]. Collimators were arranged gradually from one end of the target to the other. The inverse planning optimization process was then initiated, with dose constraints set for the target and OARs. Through quantitative evaluation of the dose-volume histogram, the spatial distribution of isocenters was refined, and optimization calculations were repeated. The final treatment plan was completed when the target coverage and OAR parameters met the clinical standards.

**Figure 1:**
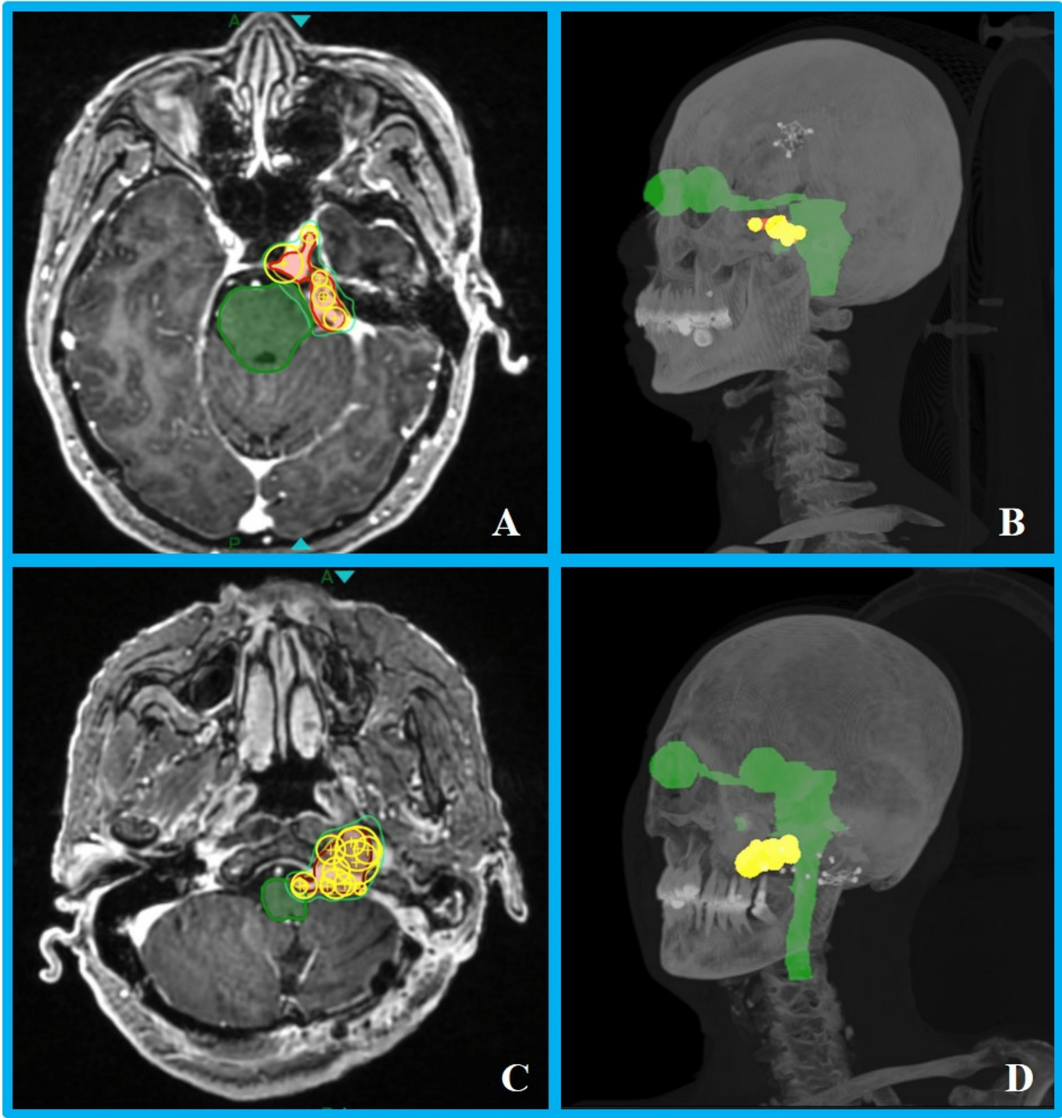
Schematic diagrams of collimator coverage for the two cases. (A) Collimator placement at the maximum axial slice of planning target volume (PTV) in Case 1. (B) 3D view of Case 1. (C) Collimator placement at the maximum axial slice of PTV in Case 2. (D) 3D view of Case 2. The red region denotes PTV, the yellow region represents the collimator sphere volume, and the green region indicates the organs at risk.

**Figure 2:**
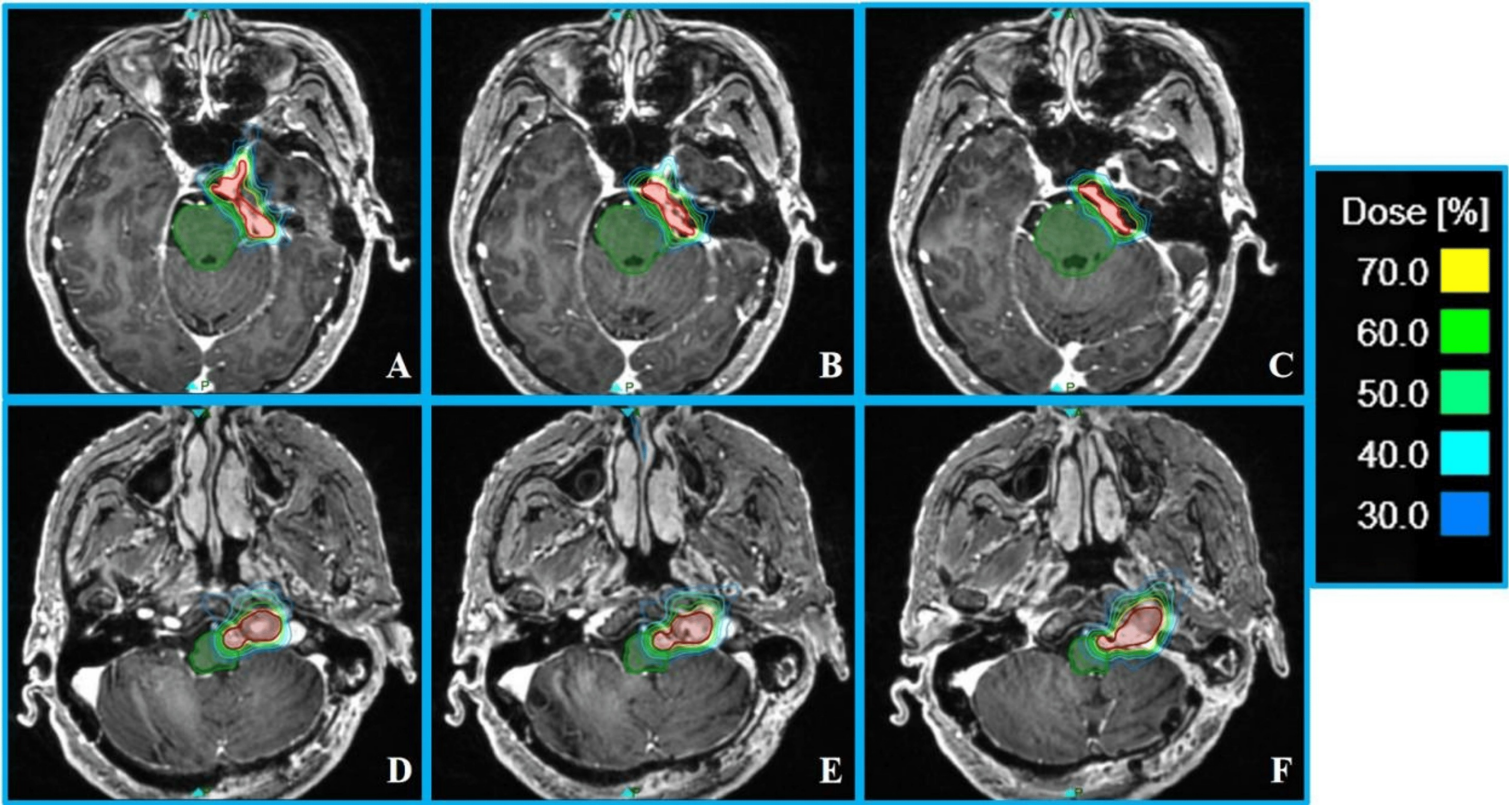
Transverse dose distribution of planning target volume (PTV) for the two cases. (A-C) Upper, middle, and lower axial sections of PTV in Case 1. (D-F) Upper, middle, and lower axial sections of PTV in Case 2. The dose lines represent a range from 70% to 30% of the prescription dose. The red region denotes the PTV, and the green region indicates the brainstem.

Case 1 utilized seven collimators (5 mm to 12.5 mm) for target coverage: one isocenter with a 12.5 mm collimator, four isocenters with 7.5 mm collimators, and two isocenters with 5 mm collimators. Larger collimators (7.5 mm and 12.5 mm) were first used for primary coverage, with 5 mm collimators filling in the edges (Figures 1A, 1B). For Case 2, 17 treatment isocenters were placed ranging from 4 mm to 15 mm: 2 × 15 mm, 1 × 12.5 mm, 6 × 10 mm, 3 × 7.5 mm, 4 × 5 mm, and 1 × 4 mm. Larger collimators (10-15 mm) were used to cover the majority volume of the target, followed by smaller collimators (4-7.5 mm) for edge filling (Figures 1C, 1D). The plan parameters, target, and OAR dosimetric results for both cases are listed in Table 1. The results showed that more irregular target shapes and larger target volumes correlated with a higher number of isocenters and longer treatment times. Despite the targets being in close proximity to the central nervous system, dosimetric parameters demonstrated that the dose to the brainstem and other OARs was safely within tolerance limits in both cases. As shown in the dose distribution (Figure 2), there was a steep dose falloff around the target, particularly in the central nervous system region, highlighting significant advantages in protecting normal brain tissue.

**Table 1:** Dosimetric parameters of the treatment plans, target volumes, and organs at risk (OARs) for the two cases. The conformity index (CI) is calculated as: CI = (TVPV × TVPV)/(TV × PV), where TVPV denotes the volume of the planning target volume (PTV) covered by the prescription dose, TV represents the total PTV volume, and PV signifies the volume encompassed by the prescription dose. The gradient index (GI) is defined as: GI = PV50%/PV, where PV50% refers to the volume covered by the 50% prescription dose, and PV is the same as defined for CI. Treatment time refers to the average single-fraction treatment duration from the start to the end of each treatment session for the patient.

		Case 1	Case 2
Isocenter		7	17
Beams		70	156
Total MU		21,773.84	23,213.39
Treatment time (minutes)		20.83	32.4
PTV	Coverage (%)	95.0%	97.6%
	CI	0.52	0.66
	GI	3.33	3.61
Brainstem	Dmax (Gy)	19.4	23.0
	V_15.9_ (cc)	0.12	0.21
Optic nerve-L	Dmax (Gy)	11.7	2.9
Optic nerve-R	Dmax (Gy)	7.8	4.4
Optic chiasm	Dmax (Gy)	10.3	4.4
Cochlea	Dmax (Gy)	6.0	9.2
Lens-L	Dmax (Gy)	0.3	3.2
Lens-R	Dmax (Gy)	2.1	0.6
Eye-L	Dmax (Gy)	5.0	3.2
Eye-R	Dmax (Gy)	6.4	5.3
Brain	V_12_ (cc)	5.08	3.21

Follow-up results

Case 1

**Figure 3:**
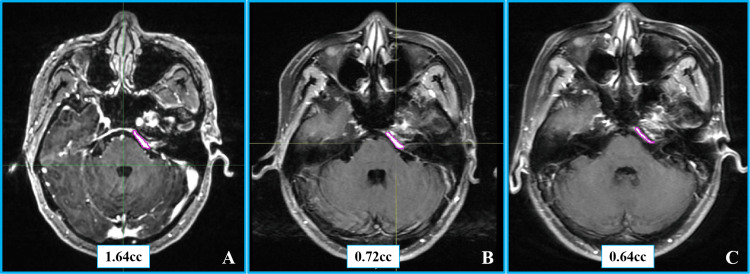
Contrast-enhanced MRI images of Case 1. (A) Axial contrast-enhanced T1-weighted image with contouring of the tumor volume before radiosurgery. (B) The same sequence with tumor volume contouring one year post-ZAP-X radiosurgery. (C) Axial contrast-enhanced T1-weighted imaging with tumor volume contouring four years post-ZAP-X radiosurgery.

On the second day of treatment, a Common Terminology Criteria for Adverse Events (CTCAE) grade 2 transient headache and vomiting occurred but resolved spontaneously. At the one-year follow-up, MRI revealed a 56.1% reduction in tumor volume (from 1.64 cm³ to 0.72 cm³), with no peritumoral edema on T2-weighted imaging. By the four-year follow-up, the tumor volume had further decreased to 0.64 cm³, representing a 61.0% reduction from the baseline (corresponding to a 15.3% annual reduction rate) and meeting the Response Assessment in Neuro-Oncology (RANO) criteria for sustained partial response [12]. No new neurological deficits or treatment-related toxicities greater than CTCAE grade 2 were observed throughout the follow-up period. Pretreatment and follow-up imaging findings are depicted in Figure 3.

Case 2

**Figure 4:**
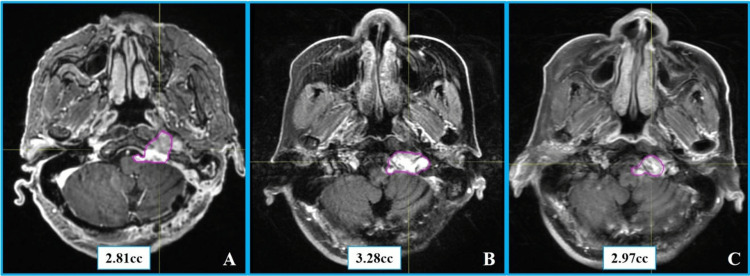
Contrast-enhanced MRI images of Case 2. (A) Axial contrast-enhanced T1-weighted imaging with contouring of the tumor volume before radiosurgery. (B) The same sequence with tumor volume contouring one year post-ZAP-X radiosurgery. (C) Axial contrast-enhanced T1-weighted imaging with tumor volume contouring three years post-ZAP-X radiosurgery.

Throughout the entire treatment course, the patient did not experience any acute radiation therapy-related toxicities of CTCAE grade ≥2. At one-year post-radiosurgery, a follow-up contrast-enhanced head MRI revealed the tumor volume to be 3.28 cm³ (representing a 16.7% increase from the baseline pretreatment volume of 2.81 cc), with no evidence of peritumoral edema on T2-weighted imaging (Edema index = 0). Continued follow-up at three years post-treatment showed stable tumor volume at 2.97 cc (a 5.7% increase from baseline), but with significantly diminished tumor enhancement on contrast-enhanced MRI. Imaging evaluation using the RANO criteria confirmed sustained stable disease. No new positive neurological signs were detected on physical examination during long-term follow-up, and treatment-related toxicity assessment indicated no adverse events of CTCAE grade ≥2. Pretreatment and follow-up imaging findings are shown in Figure 4.

## Discussion

SRS is a high-precision radiation therapy technology that delivers single or fractionated doses of focused high-dose radiation to tumors, effectively controlling tumor growth while maximizing protection of surrounding normal brain tissue. SRS has been widely used in the treatment of meningiomas, particularly for patients with inoperable or recurrent tumors. In recent years, the ZAP-X stereotactic radiosurgery system, as a novel radiosurgical device, has emerged as a new treatment option for meningiomas.

Developed by Stanford University Professor John Adler, the ZAP-X system is a fully self-shielded platform equipped with a 3 MV linear accelerator and a tungsten rotating collimator offering eight collimator sizes (4-25 mm). It delivers 3 MV X-ray to the target at a dose rate of 1,500 MU/minute with a source-to-axis distance of 450 mm. The short source-to-axis distance and innovative collimator platform reduce beam geometric penumbra, sharpening the steep dose distribution required for SRS and creating a relatively compact treatment sphere [13]. This enables high-precision radiotherapy while significantly reducing radiation doses to surrounding normal tissues, providing notable advantages in protecting critical OARs such as the optic nerve and brainstem.

This study is the first report of four-year long-term follow-up results for the ZAP-X system in treating benign meningiomas, surpassing the one to two-year short-term efficacy observations in previous studies. Through continuous tracking of these two typical cases, we found sustained improvement or stability in symptoms after treatment, with dynamic contrast-enhanced MRI showing progressive tumor volume reduction or long-term stability, and no new neurologic symptoms developed. There were no CTCAE > or = grade 2 for the second case and only transient grade 2 for the first case occurring over the 48-month observation period. Compared with internationally reported similar studies, the four-year efficacy data presented here not only validate the long-term safety of ZAP-X in meningioma treatment but, more importantly, this lends additional evidence to support its continued use. This provides critical clinical references for optimizing treatment protocols in the field of SRS for intracranial benign tumors.

Compared with traditional radiosurgical devices such as the CyberKnife, ZAP-X offers distinct advantages in dose distribution and tissue protection. In the treatment of trigeminal neuralgia, ZAP-X results in smaller irradiated volumes at the 20% and 10% isodose levels, thereby reducing exposure to adjacent key structures such as the trigeminal ganglion and brainstem, an effect that may help lower the risk of treatment-related complications [14]. Our previous institutional study [5] demonstrated that the ZAP-X system, CyberKnife G4, and Gamma Knife all meet clinical needs for SRS of single brain metastasis. The new ZAP-X platform can deliver high-quality treatment plans equivalent to or superior to those of the CyberKnife and Gamma Knife, with dose-related advantages including more conformal dose distribution and better protection of brain tissue. These advantages are of significant importance in meningioma treatment, especially for lesions near critical neurovascular structures, where precise irradiation can more effectively safeguard OARs.

Although this study preliminarily demonstrates the potential of the ZAP-X device in treating meningioma, the small sample size currently limits our ability to draw definitive conclusions about its safety and efficacy. Notably, significant research gaps exist in the long-term efficacy observation and evaluation of potential late-onset complications following treatment with this device, which require further clarification through ongoing follow-up studies. If future research can conduct multicenter randomized controlled trials, it will allow for a more precise comparison of the therapeutic differences and advantages between ZAP-X and other radiosurgical devices, thereby enabling a comprehensive and systematic assessment of its safety and efficacy in clinical application. Meanwhile, exploring the treatment adaptability of ZAP-X for meningiomas of various pathological types and anatomical locations, as well as investigating its efficacy in treating recurrent or residual meningiomas, should also be prioritized as important directions for future research in this field.

## Conclusions

In this report, we described two cases of meningioma that underwent SRS using the novel radiosurgical ZAP-X device. We discussed the clinical management, treatment processes, and follow-up outcomes of these two patients. The results indicated that neither patient developed new neurological deficits, nor did they experience adverse events of CTCAE grade ≥2. This clinical experience demonstrates that the ZAP-X system exhibits significant potential advantages in meningioma treatment, providing important practical insights and a reliable foundation for the large-scale clinical implementation of ZAP-X radiosurgical technology.
